# Risk factors for neck pain in college students: a systematic review and meta-analysis

**DOI:** 10.1186/s12889-023-16212-7

**Published:** 2023-08-08

**Authors:** Yifang Gao, Zhiming Chen, Shaoqing Chen, Shizhong Wang, Jianping Lin

**Affiliations:** 1 School of Health, Fujian Medical University, Fuzhou, China; 2 The Third Hospital of Fuqing City, Fuzhou, China; 3 College of Rehabilitation Medicine, Fujian University of Traditional Chinese Medicine, Fuzhou, China; 4 Fujian Provincial Collaborative Innovation Center of Geriatric Rehabilitation and Industry Promotion, Fuzhou, China

**Keywords:** Neck pain, College students, Risk factors, Meta-analysis

## Abstract

**Background:**

During the COVID-19 epidemic, the prevalence of neck pain among college students has increased due to the shift from offline to online learning and increasing academic and employment pressures. Therefore, this systematic review aimed to identify the personal, occupational, and psychological factors associated with the development of neck pain to promote the development of preventive strategies and early intervention treatment.

**Methods:**

Seven electronic databases were searched from inception to December 2022 for cross-sectional studies, cohort studies, case­­-­control studies, and randomized controlled trials (RCTs) on neck pain. The quality of the selected studies were assessed by American Agency for Healthcare Research and Quality (AHRQ) or the Newcastle-Ottawa Scale (NOS). Pooled odds ratios (ORs) with corresponding 95% confidence intervals (CIs) were calculated to evaluate the effects of the included risk factors on neck pain.

**Results:**

Thirty studies were included, including 18,395 participants. And a total of 33 potentially associated risk factors were identified. Ultimately, 11 risk factors were included in the meta-analysis after assessing, and all results were statistically significant (*P* < 0.05). The factors supported by strong evidence mainly include the improper use of the pillow (OR = 2.20, 95% CI: 1.39 to 3.48), lack of exercise (OR = 1.88, 95% CI: 1.53 to 2.30), improper sitting posture (OR = 1.97, 95% CI: 1.39 to 2.78), history of neck and shoulder trauma (OR = 2.32, 95% CI: 1.79 to 3.01), senior grade (OR = 2.86, 95% CI: 2.07 to 3.95), staying up late (OR = 1.80, 95% CI: 1.35 to 2.41), long-time electronic product usage daily (OR = 1.53, 95% CI: 1.33 to 1.76), long-time to bow head (OR = 2.04, 95% CI: 1.58 to 2.64), and emotional problems (OR = 2.09; 95% CI: 1.66  to 2.63). Risk factors supported by moderate evidence were high stress (OR = 1.61, 95% CI: 1.02 to 2.52) and female gender (OR = 1.69, 95% CI: 1.52 to 1.87).

**Conclusion:**

This study obtained 11 main risk factors affecting college students neck pain, including improper use of the pillow, lack of exercise, improper sitting posture, history of neck and shoulder trauma, senior grade, staying up late, long-term electronic product usage daily, long time to bow head, high stress, emotional problems and female gender.

**Supplementary Information:**

The online version contains supplementary material available at 10.1186/s12889-023-16212-7.

## Background

Neck pain is one of the most commonly reported musculoskeletal disorders, causing a substantial economic burden to healthcare systems [1], and the 2018 Global Burden of Disease report listed neck pain as one of the leading causes of long-term dysfunction [2]. In the general population, the average prevalence of neck pain is 23.1% [3], and the incidence of neck pain is very high in college students (48%-78%) [4–6]. The incidence of cervical spondylosis in college students is increasing rapidly, with an annual growth rate twice that of the 50-year-old group [7]. As studying at home became normal during the COVID-19 pandemic, the shift from offline to online learning styles seriously affected students' musculoskeletal health [8, 9]. With the emergence of online teaching, students spend more time using electronic devices to support their academic and leisure activities. Consequently, prolonged bowing at desks increases the incidence of neck pain in students [10–12]. Studies have revealed that neck pain is the leading cause of illness, decreased concentration, lower educational attainment, and college students skipping classes, thereby affecting students' future career prospects [13]. Thus, early recognition of the risk factors for neck pain is important for prevention and early intervention treatment.

The occurrence of neck pain is thought to be multifactorial, with individual, physical, and psychosocial factors that aggravate its onset and persistence [14]. Many risk factors, such as poor posture, obesity, trauma history, sex, age, and poor lifestyle, may contribute to the occurrence and development of neck pain [12, 15–17]. However, it is difficult to confirm the risk factors for neck pain in college studies owing to the unknown methodological quality. Accordingly, this meta-analysis was conducted to explore the risk factors for neck pain in college students to provide a basis for formulating preventive education and taking preventive and therapeutic measures.

## Method

### Protocol and registration

This meta-analysis was based on the Preferred Reporting Items for Systematic Reviews and Meta-analyses (PRISMA) guidelines [18] (Additional File 1). The PROSPERO registration number for this systematic review is CRD42022333624.

### Search strategy

The databases PubMed, Web of Science, Cochrane Library, Embase database, China National Knowledge Information Database (CNKI), WanFang Database, and Chinese Scientific Journal Database (VIP) were systematically and independently searched from databases establishment to December 2022 using the following MeSH terms and all related free search terms: (((((((((((((((neck pain[MeSH Terms]) OR (neck pain)) OR (neck discomfort[MeSH Terms])) OR (neck discomfort)) OR (neck ache[MeSH Terms])) OR (neck ache)) OR (cervicalgia[MeSH Terms])) OR (cervicalgia)) OR (nuchal pain[MeSH Terms])) OR (nuchal pain)) OR (cervical region discomfort[MeSH Terms])) OR (cervical region discomfort)) OR (cervical spondylosis[MeSH Terms])) OR (cervical spondylosis)) AND ((factor[MeSH Terms]) OR (factor))) AND ((((((((((((college students[MeSH Terms]) OR (college students)) OR (university students[MeSH Terms])) OR (university students)) OR (young adults[MeSH Terms])) OR (young adults)) OR (higher education students[MeSH Terms])) OR (higher education students)) OR (undergraduates[MeSH Terms])) OR (undergraduates)) OR (academic learners[MeSH Terms])) OR (academic learners)).

### Inclusion and exclusion criteria

The titles and abstracts were first screened, and then the full text of the eligible literature was further examined independently by two reviewers. Any disagreement in the selection process was resolved through a full consultation with a third reviewer. Studies included in this meta-analysis met the following criteria: (1) cross-sectional, cohort studies, case–control studies, or RCTs (2) studies with original clear OR values (Odds Ratio) and 95% confidence interval (CI) that could be extracted or calculated with OR values and 95% CI (3) research subjects were college students, and (4) studies published in Chinese or English. The following studies were excluded: (1) studies that lacked data or did not analyze factors (2) full texts were unavailable (3) non-journal articles such as dissertations and conference papers, and (4) republished studies.

### Data extraction and quality assessment

Two reviewers independently extracted the following information: first author, publication year, geographic region, study design, sample size, mean age, relevant risk factors, and OR (95% CI). The same two reviewers independently used the American Agency for Healthcare Research and Quality (AHRQ) to evaluate cross-sectional studies and the Newcastle-Ottawa Scale (NOS) to evaluate case-control and cohort studies [19, 20]. The AHRQ included 11 items that answered "yes,” "no, " or "unclear" [21]. If the answer is "no" or "unclear,” the score is 0, and if the answer is "yes,” a score of 1-3 indicates low quality, 4-7 indicated medium quality and 8-11 indicated high quality. The NOS included eight items in three blocks: subject selection, comparability between groups, and outcome measurement. The full score was 9, with 0-4 being low quality, 5-6 being moderate quality, and 7-9 being high quality. Any disagreement regarding data extraction or quality assessment was resolved through a full discussion with a third reviewer.

### Strength of evidence

To determine the level of evidence for each influencing factor, and based on the quality of the studies, the existing evidence scales were used for assessment [22, 23] and were defined as follows: (1) strong evidence: the results come from a pool of three or more studies, at least two of which are high-quality homogeneous studies or synthesis of multiple high-quality studies (2) moderate evidence: statistically significant results from a combination of one high-quality study and one or more studies of moderate or low quality (3) limited evidence: the results come from a high-quality study or a combination of one or more moderate or low-quality studies, and (4) very limited evidence: the no evidence: significantly pooled results from multiple studies where heterogeneity findings were unrelated to quality.

### Statistical analysis

Articles were grouped according to the type of risk factor using forest plots presenting results for the same factors. To ensure the reliability of the pooled effect estimates size, we only performed a meta-analysis of the risk factors assessed in at least three different studies. Data from two or fewer studies or factors with different results were presented in tables without summary analysis [24, 25]. Pooled ORs with corresponding 95% CIs were calculated to estimate the effect of risk factors on the occurrence of neck pain. If there were no OR values, the software was used for conversion. Heterogeneity across all included studies was assessed and quantified using Cochrane Q statistics and I^2^ statistics [26]. The greater the I^2^ value, the greater the heterogeneity. The low, moderate, and high degrees of heterogeneity were represented by I^2^ statistics of 25%, 50%, and 75%, respectively [27]. For results with high heterogeneity, sensitivity analysis was performed to determine the stability of the conclusions by excluding each study from the meta-analysis [28]. Funnel plot analysis was performed when at least ten studies were included in the analysis [29].

## Results

### Study selection

**Fig. 1:**
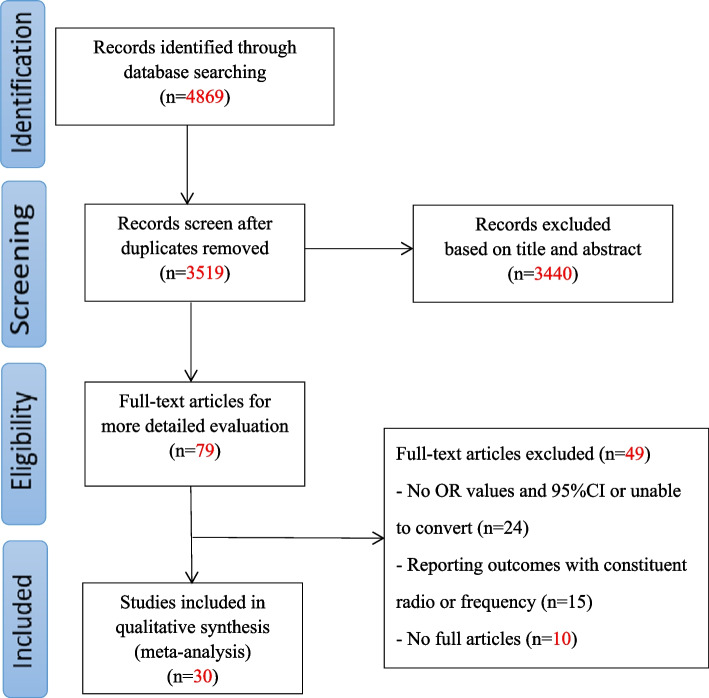
Flowchart of the search process for the articles

A total of 4869 related studies were identified by searching seven electronic databases, which were reduced to 3519 articles after removing duplicates; 3440 were excluded after reading the titles and abstracts. The reasons for exclusion included reviews, dissertations, conference papers, and research objects other than college students. After carefully reading the full text of the remaining 79 articles, 46 studies were excluded for the following reasons: (1) a lack of OR values and 95% CI or unable to convert (n = 24) (2) reporting outcomes with constituent ratio or frequency (n = 15), and (3) no full-text articles (n = 10). Finally, 30 studies were included, with 15 in English and 15 in Chinese. A PRISMA flow diagram was illustrated in Fig. 1.

### Study characteristics and quality assessment

The basic characteristics of the included studies and quality assessment results were summarized in Table 1. A total of 30 studies were included [12, 30–58], including 24 cross-sectional studies, 2 cohort studies, and 4 case-control studies. Of these, 16 were rated as high quality and 14 as medium quality, with an overall average score of 7.67. The overall quality of the study was good.

**Table 1:** Baseline characteristics of all included studies in our meta-analysis

Literature	Publication (Year)	Geographic region	Study design	Sample size	Mean age	Risk factors reported	Quality score
Li YW [30]	2020	China	Cross-sectional study	4848	19. 16 ± 1. 39	4、13、14	7
Hu YY [31]	2018	China	Cross-sectional study	392	NR	2、3、7	8
Meng FY [32]	2020	China	Cross-sectional study	836	20.15 ± 1.96	9、13	9
Ye F [33]	2016	China	Cross-sectional study	1117	20.62 ± 1.266	4、14、17、20	8
Sun Z [34]	2019	China	Cross-sectional study	448	19.23 ± 1.05	2、3、5、15、21、22	6
Su JT [35]	2013	China	Case–control study	NG:180 CG:180	NG:23.77 ± 2.44 CG:23.62 ± 2.33	1、3、9、12、14、20、23	8
Zhu XTg [36]	2021	China	Cross-sectional study	358	19. 91 ± 1. 13	1、3、6、9、12、13、16	8
Ren YC [37]	2013	China	Cross-sectional study	2026	NR	3、13、14、17、24	7
Wang CL [38]	2014	China	Cross-sectional study	303	NR	12、24	7
Tian ZY [39]	2019	China	Cross-sectional study	478	NR	1、2、3、6、14、29	6
Jing LN [40]	2021	China	Cross-sectional study	803	20.40 ± 1.30	2、14、18	8
Wang Z [41]	2021	China	Cross-sectional study	408	19.54 ± 1.066	6、13、25	6
Hashim R [42]	2021	United Arab Emirates	Cross-sectional study	202	NR	6	7
Ayhualem S [43]	2021	Ethiopia	Cross-sectional study	808	21.9 ± 2.15	7、9、13、26、27、30	8
Daher A [44]	2021	Israel	Cross-sectional study	295	27.7 ± 8.32	15、16	6
Behera P [45]	2020	India	Cross-sectional study	331	20.5 ± 1.7	7、8、14	7
Weleslassie GG [12]	2020	Ethiopia	Cross-sectional study	419	22 ± 2.215	8、9、14、25	8
Dighriri YH [46]	2019	Saudi Arabia	Cross-sectional study	440	22.4 ± 1.6	6、11、12	8
Haroon H [47]	2018	Pakistan	Cross-sectional study	360	20.77 ± 1.47	6、13、15	8
Algarni AD [48]	2017	Saudi Arabia	Cross-sectional study	469	21.4 ± 1.3	6、11、12	7
Alshagga MA [49]	2013	Malaysia	Cross-sectional study	232	20.6 ± 2.2	6、7、10、13	7
Ndetan HT [50]	2009	Dallas	Cross-sectional study	252	25.6 ± 4.5	10	8
Kanchanomai S [51]	2011	Thailand	Cohort study	524	19.4 ± 1.1	7、19、28	8
Liu HT [52]	2014	China	Case–control study	NG:66 CG:66	NR	14	8
Zhang JL [53]	2009	China	Case–control study	NG:120 CG:120	NG:20.03 ± 0.41 CG:21.01 ± 0.42	3、13、14	8
Huang ZH [54]	2016	China	Case–control study	NG:111 CG:337	19.2 ± 1.0	2、5	6
Lin Y [55]	2022	China	Cross-sectional study	1178	21.1 ± 1.7	4、15、31	7
Wah SW [56]	2022	Myanmar	Cross-sectional study	81	NR	13、32	7
Puntumetakul R [57]	2022	Thailand	Cross-sectional study	237	20.54 ± 1.35	13	8
Hodačová L [58]	2022	The Czech Republic	Cohort study	73	NR	33	8

Risk factors: 1-improper use of the pillow; 2-staying up late; 3-improper sitting posture; 4-female gender; 5-frequent alcohol consumption; 6-history of neck and shoulder trauma; 7-senior grade; 8-history of pain; 9-lack of exercise; 10-obesity; 11-history of psychosomatic symptoms; 12-emotional problems; 13-long-time electronic product usage daily; 14-long-time to bow head; 15-high stress; 16-sedentariness; 17-poor sleep quality; 18-throat inflammation; 19-improper keyboard position; 20-heavy schoolbag; 21-age; 22-sleeping on a bus or car; 23-feeling cold and wet wind; 24-neck fatigue; 25-poor head and neck posture; 26-cigarette smoking; 27-lack of rest; 28-computer screen is not positioned at a level horizontal with the eyes; 29-desks and chairs not matching height; 30-many social media; 31-major; 32-the years of smartphone use; 33-top-level sport. *NG* neck pain group, *CG* control group, *NR* not reported

### Risk factors for neck pain in college students

Overall, 33 potential risk factors were extracted from the 30 eligible studies, including improper use of the pillow, staying up late, improper sitting posture, female gender, frequent alcohol consumption, history of neck and shoulder trauma, senior grade, history of pain, lack of exercise, obesity, history of psychosomatic symptoms, emotional problems, long-time electronic product usage daily, long-time to bow head, high stress, sedentariness, poor sleep quality, throat inflammation, improper keyboard position, heavy schoolbag, age, sleeping on a bus or car, feeling cold and wet wind, neck fatigue, poor head and neck posture, cigarette smoking, lack of rest, computer screen is not positioned at a level horizontal with the eyes, desks and chairs not matching height, many social media, major, the years of smartphone use and top-level sport. There were eleven risk factors, including improper use of the pillow, lack of exercise, improper sitting posture, history of neck and shoulder trauma, senior grade, staying up late, long time electronic product usage daily, long time to bow head, high stress, emotional problems and female gender that met the criteria for inclusion in the meta-analysis. The combined results and evidence levels are presented in Table 2.

**Table 2:** Risk factors' pooled analysis and level of evidence

Risk factors	Number of included studies	Pooled effects			Level of evidence
		**0)** **OR[95%CI]**	***P* value**	I^2^	
**Improper use of the pillow**	3	2.20 [1.39, 3.48]	0.0008	66%	Strong
**Lack of exercise**	5	1.88 [1.53, 2.30]	< 0.00001	0%	Strong
**Improper sitting posture**	7	1.97 [1.39, 2.78]	0.0001	78%	Strong
**History of neck and shoulder trauma**	8	2.32 [1.79, 3.01]	< 0.00001	22%	Strong
**Senior grade**	5	2.86 [2.07, 3.95]	< 0.00001	5%	Strong
**Staying up late**	6	1.80 [1.35, 2.41]	< 0.0001	55%	Strong
**Long-time electronic product usage per day**	11	1.53 [1.33, 1.76]	< 0.00001	78%	Strong
**Long-time to bow head**	10	2.04 [1.58, 2.64]	< 0.00001	82%	Strong
**High stress**	4	1.61 [1.02, 2.52]	0.04	88%	Moderate
**Emotional problems**	5	2.09 [1.66, 2.63]	< 0.00001	0%	Strong
**Female gender**	3	1.69 [1.52, 1.87]	< 0.00001	0%	Moderate

### Improper use of the pillow

**Fig. 2:**
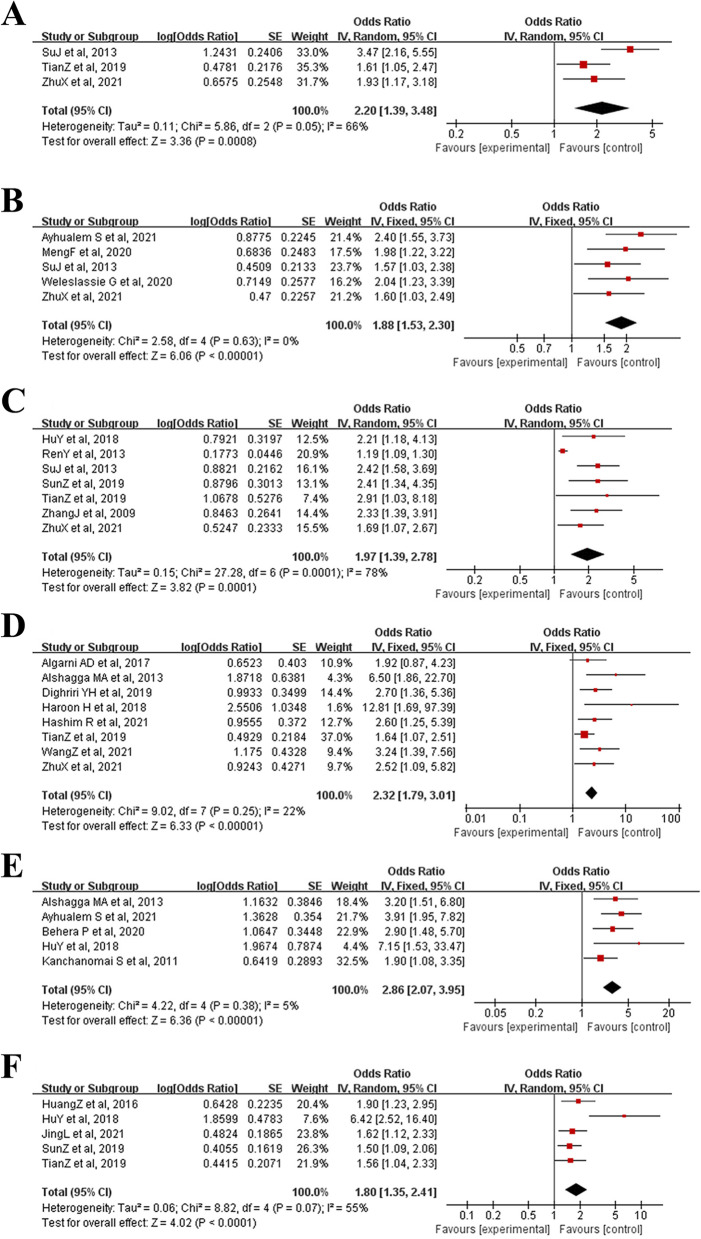

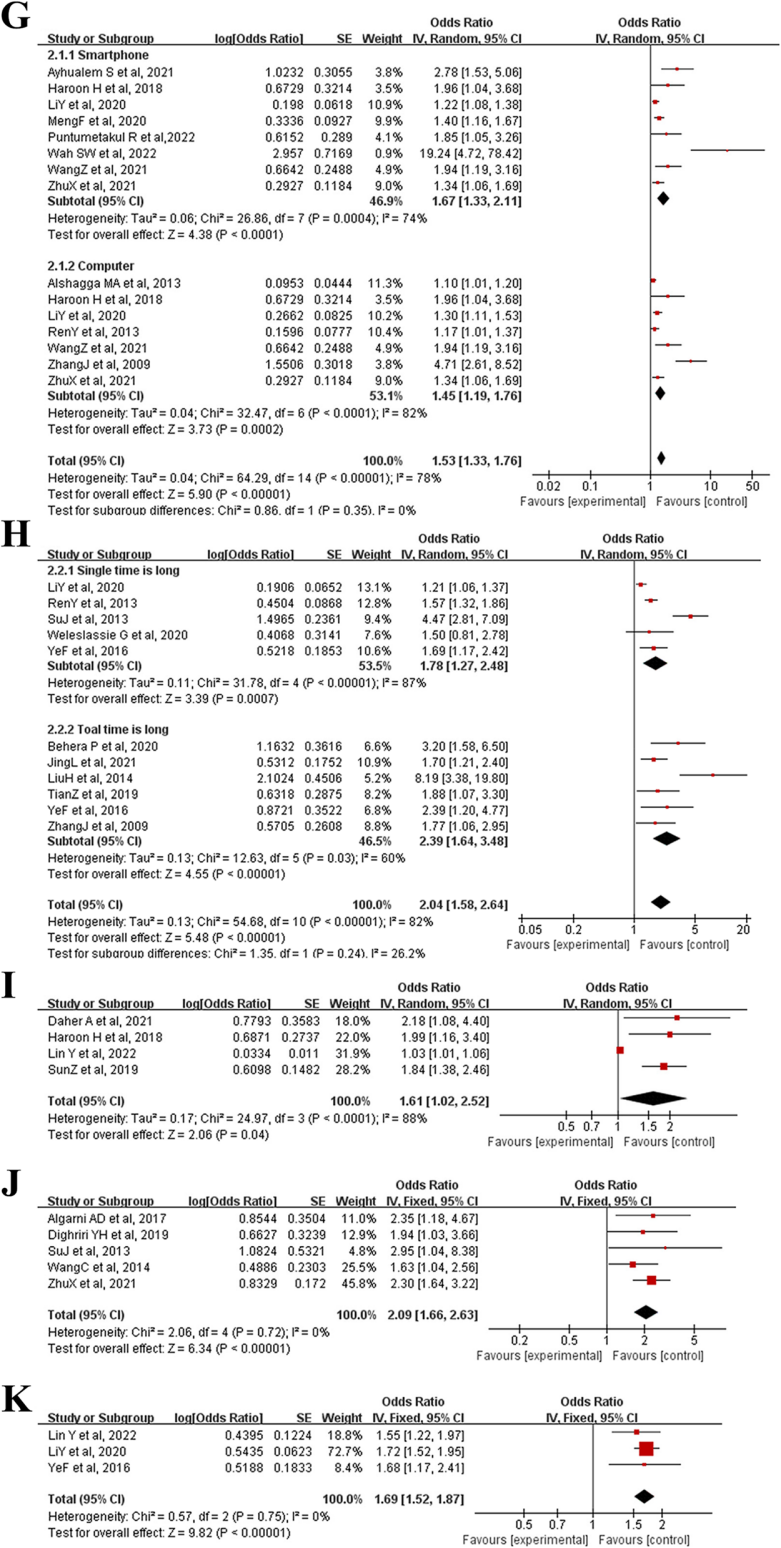
Meta-analysis of risk factors for neck pain in college students (A:Improper use of the pillow, B:Lack of exercise, C:Improper sitting posture, D:History of neck and shoulder trauma, E:Senior grade, F:Staying up late, G:Long-time electronic product usage per day, H:Long-time to bow head, I:High stress, J:Emotional problems, K:Female gender)

Three studies [35, 36, 39] reported the effect of improper use of pillows on neck pain in college students, investigating a total of 1016 participants. Our results depicted that improper use of the pillow was one of the risk factors for neck pain in college students (OR = 2.20, 95% CI: 1.39 to 3.48, *P* = 0.0008). However, because the heterogeneity between studies was high (I^2^ = 66%) (Fig. 2A), a sensitivity analysis was performed. When excluding study of SuJ et al., 2013, the heterogeneity decreased significantly (I^2^ = 0%) (Additional File 2: Supplementary figure A), while the study conclusions remained statistically significant (*P* < 0.05).

### Lack of exercise

Compared to regular exercise, five studies [12, 32, 35, 36, 43] found that physical inactivity was associated with a higher prevalence of neck pain in college students, with minimal heterogeneity (OR = 1.88, 95% CI: 1.53 to 2.30, *P* < 0.00001, I^2^ = 0%) (Fig. 2B).

### Improper sitting posture

Seven studies [31, 34–37, 39, 53] analyzed the influence of improper sitting posture on neck pain in college students, investigated all 4002 participants, and found evidence of a positive correlation between this factor and neck pain. The combined analysis results revealed that improper sitting posture caused statistically significant harm to neck pain (OR = 1.97, 95% CI: 1.39 to 2.78, *P* = 0.0001). However, due to the high heterogeneity between the studies (I^2^ = 78%) (Fig. 2C), a sensitivity analysis was performed. When excluding the study by RenY et al., 2013, the heterogeneity decreased significantly (I^2^ = 0%) (Additional File 2: Supplementary figure B), while the study conclusions remained statistically significant (*P* < 0.05).

### History of neck and shoulder trauma

Eight studies [36, 39, 41, 42, 46–49] investigated all 2947 participants, and all reported the impact of a history of neck and shoulder disease on neck pain in college students. Our pooled results demonstrated that college students with a history of neck and shoulder trauma were more likely to develop neck pain, with a high prevalence and less heterogeneity (OR = 2.32, 95% CI: 1.79 to 3.01, *P* < 0.00001, I^2^ = 22%) (Fig. 2D).

### Senior grade

Five studies [31, 43, 45, 49, 51] analyzed the effect of grade differences on neck pain among 2287 college students. The combined results depicted that a higher probability and frequency of neck pain occurred at grade level (OR = 2.86, 95% CI: 2.07 to 3.95,* P* < 0.00001, I^2^ = 5%) (Fig. 2E).

### Staying up late

Five studies [31, 34, 39, 40, 54] that evaluated the association between staying up late and neck pain in college students were included in the meta-analysis. The combined results revealed that a high prevalence of neck pain in college students was associated with often staying up late (OR = 1.80, 95% CI: 1.35 to 2.41, *P* < 0.0001). However, due to its high heterogeneity (I^2^ = 55%) (Fig. 2F), it was reduced by excluding the articles one by one. Finally, the heterogeneity decreased significantly when excluding HuY et al., 2018 (I^2^ = 0%) (Additional File 2: Supplementary figure C), while the results remained statistically significant (*P* < 0.00001).

### Long-time electronic product usage per day

**Fig. 3:**
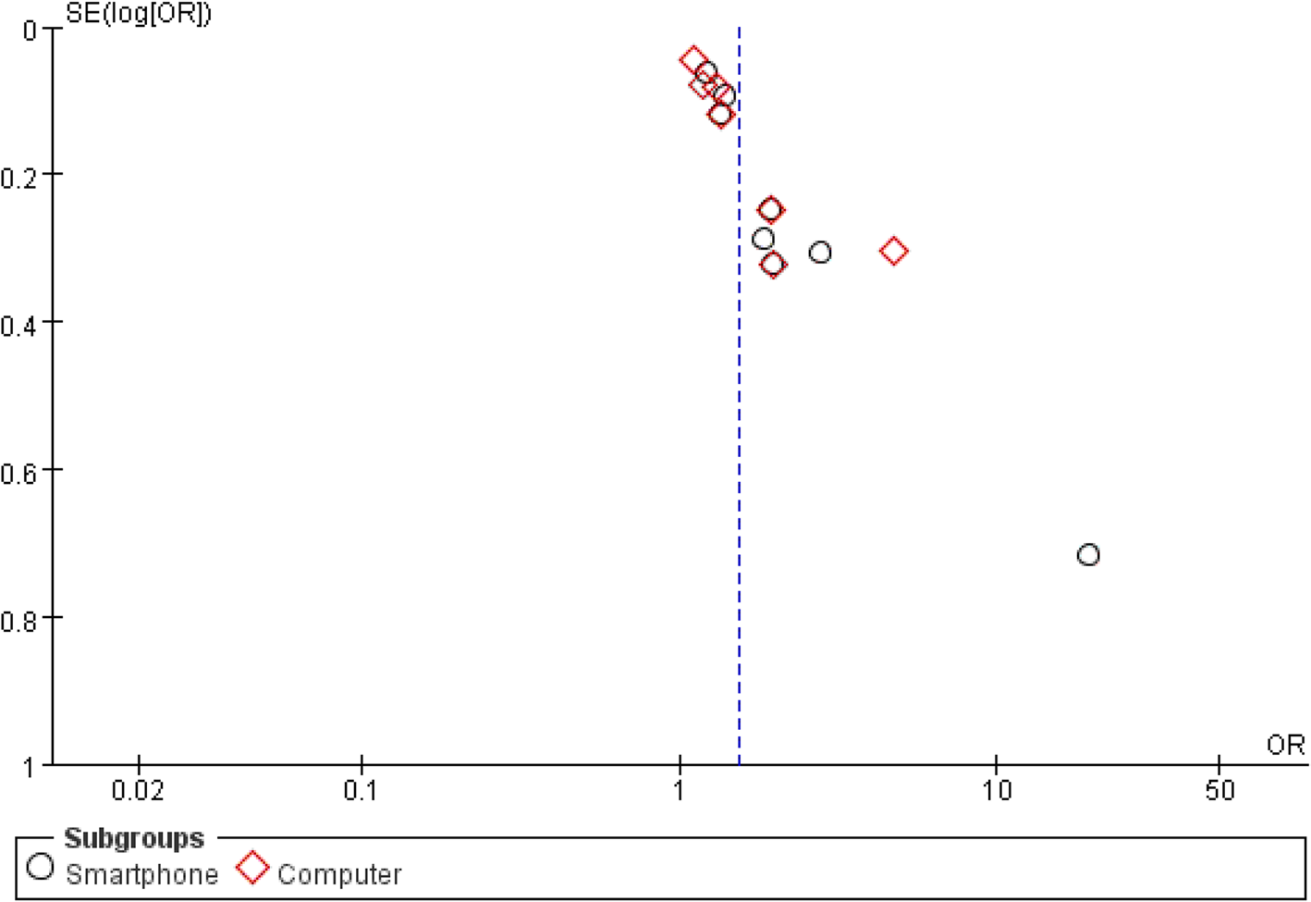
Funnel plot about meta-analysis of risk factors about long-time electronic product usage per day

Eleven studies [30, 32, 36, 37, 41, 43, 47, 49, 53, 56, 57] analyzed the impact of a long-time electronic product used daily on neck pain in 10,314 college students. The results of the combined meta-analysis demonstrated that prolonged use of electronics was significantly associated with neck pain in college students (OR = 1.53, 95% CI: 1.33 to 1.76, *P* < 0.00001). Subgroup meta-analysis revealed that both smartphone and computer use could cause neck pain in college students (*P* < 0.0001 and *P* = 0.0002, respectively), but due to the combined heterogeneity (I^2^ = 78%) (Fig. 2G), the sensitivity analysis found that the overall heterogeneity was still relatively large regardless of which study was removed. The funnel plot shows almost bilateral symmetry and is less likely to be influenced by publication bias (Fig. 3).

### Long-time to bow head

**Fig. 4:**
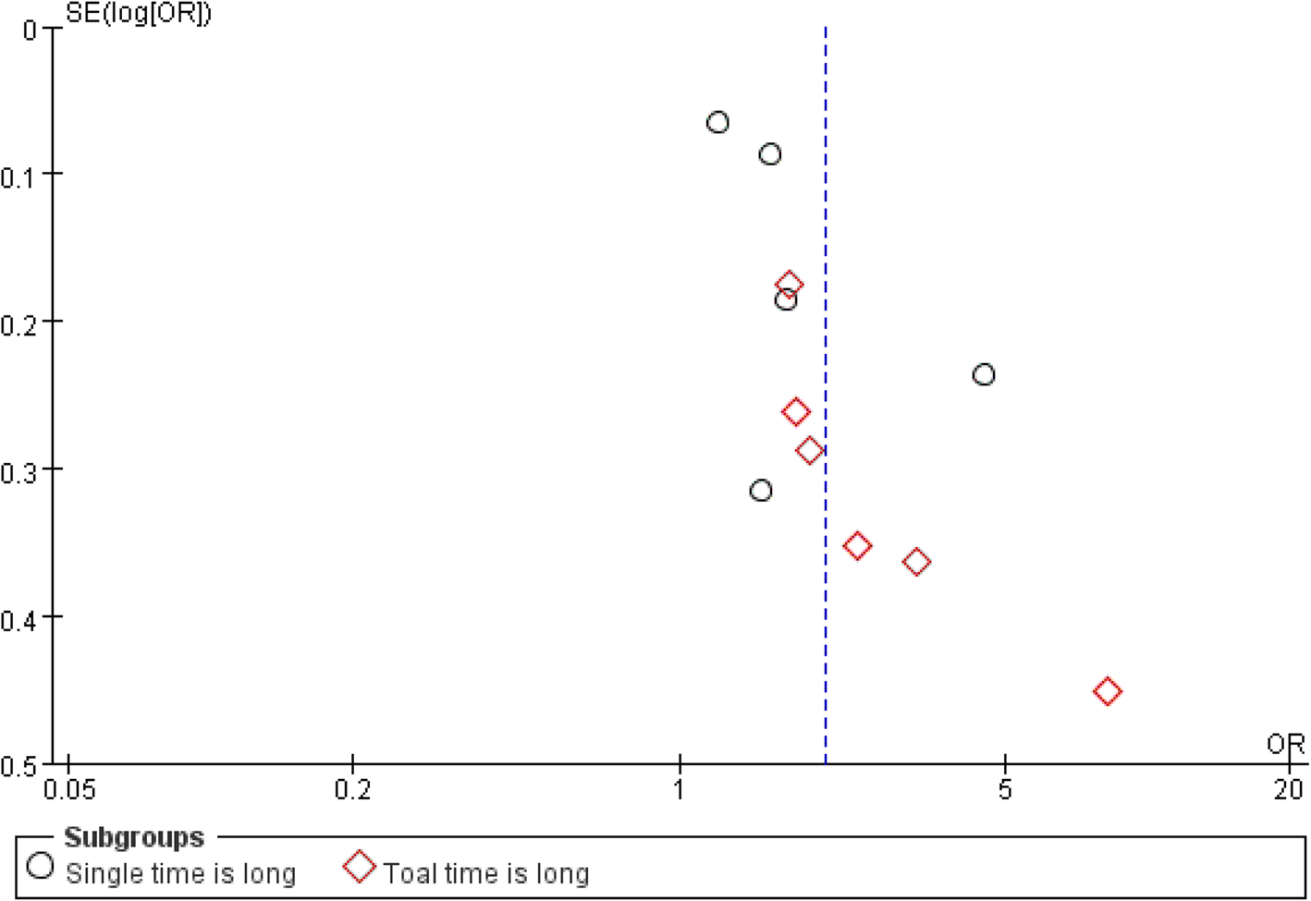
Funnel plot about meta-analysis of risk factors about long-time to bow head

Ten studies [12, 30, 33, 35, 37, 39, 40, 45, 52, 53] reported the impact of long bowing on neck pain in college students, investigating a total of 10,388 participants. The summary results demonstrated that a long time to bow head would lead to frequent neck pain among college students (OR = 2.04, 95% CI: 1.58 to 2.64, *P* < 0.00001). The results of the subgroup analysis revealed that both a single time and total time to bow the head had a certain impact on neck pain in college students (*P* = 0.0007 and *P* < 0.00001). However, due to the large heterogeneity of the combined results (I^2^ = 82%), the heterogeneity could not be reduced by excluding studies individually (Fig. 2H). The funnel plot depicted bilateral asymmetries, which may have a publication bias (Fig. 4).

### High stress

Four studies [34, 44, 47, 55] analyzed the effects of high stress on neck pain among college students. The combined results showed that the greater the stress, the more likely they were to cause neck pain (OR = 1.61, 95% CI: 1.02 to 2.52, *P* < 0.00001, I^2^ = 88%) (Fig. 2I). Due to the large heterogeneity, sensitivity analysis found that the heterogeneity decreased significantly when excluding the study of Lin Y et al., 2022 (I^2^ = 0%) (Additional File 2: Supplementary figure D), and the results remained statistically significant (*P* < 0.00001).

### Emotional problems

Five studies [35, 36, 38, 46, 48] that evaluated the relationship between emotional problems and neck pain in college students were included in the meta-analysis. The combined results demonstrated that college students with emotional problems were more likely to have neck pain than happy people (OR = 2.09; 95% CI: 1.66,2.63, *P* < 0.00001, I^2^ = 0%) (Fig. 2J).

#### Female gender

Three studies [30, 33, 55] concluded that female gender are one of the risk factors for neck pain in college students. The comprehensive analysis results showed that the prevalence of neck pain among female gender was much higher than that of male students (OR = 1.69; 95% CI: 1.52,1.87, *P* < 0.00001, I^2^ = 0%) (Fig. 2K).

#### Other risk factors

Although other risk factors, such as age, obesity, and frequent alcohol consumption, were found in their respective studies to be neck pain among college students, it was worth noting that these factors were only evaluated in one or two studies, indicating that there may not be enough evidence to prove the association with neck pain. Therefore, they are not in summary analysis, but the data was presented in the form of a table (Additional File 3).

## Discussion

This study explored the risk factors for neck pain in college students and conducted a meta-analysis of 11 risk factors, including 30 studies. Meta-analysis revealed that 11 risk factors were closely related to neck pain in college students. Although the remaining 22 factors also revealed a significant association with the occurrence of neck pain, they were not included in the meta-analysis due to the limited number of studies. There were eight previous reviews [1, 23, 59–64] and four meta-analyses [16, 65–67] on the risk factors of neck pain. However, the eight reviews lacked quantitative analysis, and the four meta-analyses included only English studies. Furthermore, the study subjects were not college students but mainly office workers, fighter aircrews, and so on [1, 16, 23, 59–67]. Only one study [65] included young people aged 18–29 years, which may have involved a small proportion of college students. In addition, the research results depicted that depression, physical inactivity, history of trauma, female gender, prolonged computer use, bowing your head for a long time at your desk, obesity, and incorrect sitting posture were closely related to the occurrence of neck pain, similar to the results of the present study results [1, 16, 23, 59, 60, 62, 63]. However, there are a few studies that are inconsistent with the results of this study. Jun et al. [65, 66] found that the duration of computer use, continuous head bowing, and obesity were not associated with the occurrence of neck pain. Ariëns et al. [64] found that sedentariness was also an important risk factor for neck pain. This may be due to the small sample size of these three studies, which was insufficient to explain the correlation with the occurrence of neck pain.

Zhu et al. demonstrated that improper pillow use is mainly related to improper pillow height [36, 39], while appropriate height, moderate softness, and hardness could prevent neck discomfort. A previous systematic review and meta-analysis reflected exercise as a protective factor and reported a negative correlation between physical activity and musculoskeletal problems [68]. Ren et al. revealed that exercise time < 3 h per week [36, 37] or exercise frequency ≤ 5 times per week [35] could cause neck pain. Therefore, regular and moderate exercise could be used as a measure to prevent neck diseases [69, 70]. Improper sitting posture is mainly manifested in jittering and bumping of the legs or crossed legs [34], incorrect sitting posture [36, 39], and head-neck lateral deviation, flexion, and rotation [37] when sitting. This affects the dynamic and static balance of the neck and causes neck pain [71]. Studies have indicated that common histories of neck and shoulder trauma mainly include discomfort, such as being directly hit, head landing when falling from a height, sprains, or experiencing pain and stiffness of the head and neck due to a sudden brake [41]. Trauma can cause damage to the neck muscles, tendons, fascia, and ligaments, which can impact the cervical spine [72]. The high incidence of neck pain among college students is also partly due to the heavy workload and pressures of work and postgraduate entrance examinations, as students may stay up late more frequently, and excessive working hours may cause deformation of the neck and shoulders, leading to soft tissue damage and increasing the risk of neck pain [6, 48].

Studies have revealed that total daily electronic product use of ≥ 3 h and a long time to bow your head ≥ 1 h is likely to lead to neck pain [12, 30, 33, 36, 40, 43, 45, 47, 56, 57]^.^ Therefore, with the increased use of electronic products and the academic burden, the prevalence of neck pain among college students is also increasing.

Among emotional problems, the most common risk factor for neck pain was depressive symptoms. Several studies have demonstrated that neck pain in college students is closely associated with a history of depression [35, 38, 46, 48]. Moreover, feeling low or nervous frequently is also a major risk factor for neck disease in college students [36]. It is clear that the influence of psychological factors on neck diseases may be the same as that of physical risk factors, and there is a significant positive correlation with neck pain [16].

## Strengthens and limitations

This is the first systematic review and meta-analysis of risk factors for neck pain in college students to specifically address and prevent the development of risk factors for college students with neck pain. This review summarized the results of 30 prospective studies, the quality of which was moderate or above.

However, this systematic review and meta-analysis had some limitations. Firstly, we only included studies published in English and Chinese, and there may be potential publication and language biases; therefore, future studies should include a wider range of languages. Secondly, most included studies were cross-sectional, so no causal relationship between exposure factors and outcomes was established, and recall bias is likely to exist. Thirdly, there is no consensus on the definition of neck pain duration ranging from 3 to 12 months. Fourthly, most of the included studies only gave the risk factors without specific evaluation criteria. Finaly, some of the studies were conducted online, so it may exist self-selection or volunteer bias.

## Conclusion

To sum up, although there were many studies in investigating the risk factors of neck pain, and found a large number of risk factors, but our study summarized eleven strong evidence risk factors, including the improper use of the pillow, lack of exercise, improper sitting posture, a history of neck and shoulder trauma, senior grade, staying up late, female gender, long time electronic product usage daily, long time to bow head, high stress and emotional problems. These eleven risk factors may lay a foundation for future neck disease prevention. However, there were still many factors that couldn't conduct meta-analysis due to few included studies. Therefore, it is recommended that more multicente and large-sample original studies closely related to these 11 risk factors could be conducted in the future to provide early warning for clinical practice and prevent neck pain in college students.

### Supplementary Information

**Additional file 1:** **Supplementary table 1.** PRISMA check list .

**Additional file 2:** **Supplementary figure 1.** Sensitivity analysis of risk factors for neck pain in college students (A:Improper use of the pillow, B:Improper sitting posture, C:Staying up late, D:High stress).

**Additional file 3:** **Supplementary table 2.** Other risk factors for neck pain in college students.

## Data Availability

All data are included in this manuscript.

## Abbreviations

OR: Odds Ratio
95% CI: 95% Confidence Interval
AHRQ: American Agency for Healthcare Research and Quality
NOS: Newcastle-Ottawa Scale
RCT: Randomized Controlled Trial
CNKI: China National Knowledge Information Database
PRISMA: Preferred Reporting Items for Systematic Reviews and Meta-Analyses
