# Supplementary material for: Risk factors for neck pain in college students: a systematic review and meta-analysis
Source: BMC Public Health. 2023 Aug 8;23:1502. doi: 10.1186/s12889-023-16212-7 (PMC10408143; doi:10.1186/s12889-023-16212-7)
Supplement: Supplementary file 2 — Additional file 2: Supplementary figure 1. Sensitivity analysis of risk factors for neck pain in college students (A:Improper use of the pillow, B:Improper sitting posture, C:Staying up late, D:High stress). [file 12889_2023_16212_MOESM2_ESM.docx]

| 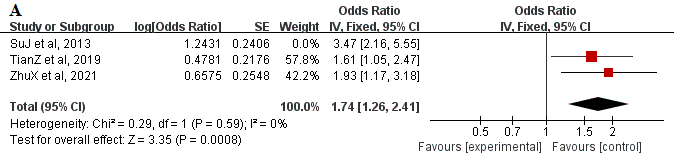 |
| --- |
| 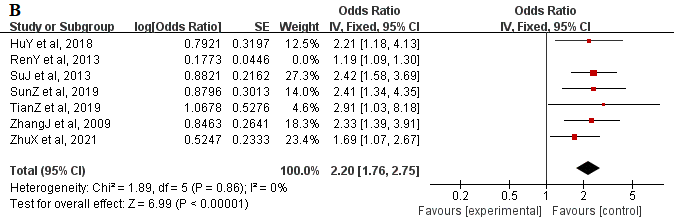 |
| 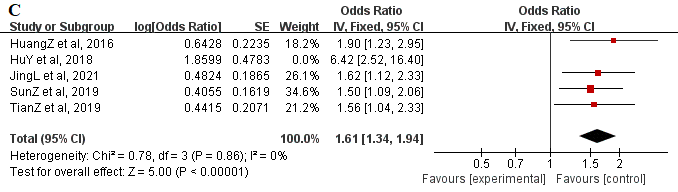 |
| 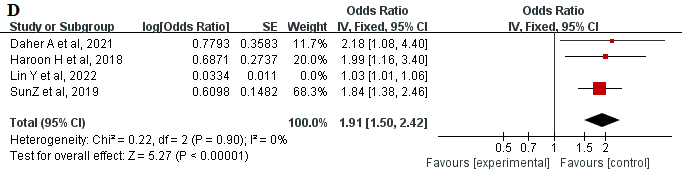 |
| **Supplementary figure 1.** Sensitivity analysis of risk factors for neck pain in college students (A:Improper use of the pillow, B:Improper sitting posture, C:Staying up late, D:High stress) |
