# Supplementary material for: Risk factors for neck pain in college students: a systematic review and meta-analysis
Source: BMC Public Health. 2023 Aug 8;23:1502. doi: 10.1186/s12889-023-16212-7 (PMC10408143; doi:10.1186/s12889-023-16212-7)
Supplement: Supplementary file 3 — Additional file 3: Supplementary table 2. Other risk factors for neck pain in college students. [file 12889_2023_16212_MOESM3_ESM.docx]

| **Supplementary table 2.** Other risk factors for neck pain in college students | | | |
| --- | --- | --- | --- |
| **Risk factor** | **Studies** | **OR** | **95%CI** |
| Frequent alcohol consumption | Huang ZH et al,2016 | 3.908 | 1.750，8.732 |
|  | Sun Z et al,2019 | 1.74 | 1.06, 2.85 |
| History of pain | Behera P et al,2020 | 13.70 | 7.60, 24.70 |
|  | Weleslassie GG et al,2020 | 11.811 | 5.460, 25.549 |
| Obesity | Alshagga MA et al,2013 | 1.10 | 1.00, 1.10 |
|  | Ndetan HT et al,2009 | 1.50 | 0.90, 2.57 |
| History of psychosomatic symptom | Dighriri YH et al,2019 | 2.98 | 1.71, 5.18 |
|  | Algarni AD et al,2017 | 2.69 | 1.37, 5.27 |
| Sedentariness | Daher A et al,2021 | 4.07 | 1.74, 9.50 |
|  | Zhu XT et al,2021 | 1.32 | 1.03, 1.70 |
| Poor sleep quality | Ren YC et al,2013 | 1.590 | 1.393, 1.816 |
|  | Ye F et al,2016 | 4.547 | 1.807, 11.440 |
| Throat inflammation | Jing LN et al,2021 | 1.803 | 1.189, 2.734 |
| Improper keyboard position | Kanchanomai S et al,2011 | 2.18 | 1.21, 3.91 |
| Heavy schoolbag | Su JT et al,2013 | 1.586 | 1.039, 2.421 |
|  | Ye F et al,2016 | 2.792 | 1.170, 6.665 |
| Age | Sun Z et al,2019 | 1.23 | 1.04, 1.45 |
| Sleeping on a bus or car | Sun Z et al,2019 | 1.52 | 1.08, 2.14 |
| Feeling cold and wet wind | SuJ et al,2013 | 2.144 | 1.345, 3.416 |
| Neck fatigue | Ren YC et al,2013 | 1.259 | 1.059, 1.498 |
|  | Wang CL et al,2014 | 1.183 | 1.025, 1.365 |
| Poor head and neck posture | Weleslassie GG et al,2020 | 3.871 | 2.311, 6.484 |
|  | Wang Z et al,2021 | 2.788 | 1.533, 5.068 |
| Cigarette smoking | Ayhualem S et al,2021 | 5.415 | 2.685, 10.919 |
| Lack of rest | Ayhualem S et al,2021 | 3.253 | 2.252, 4.699 |
| Computer screen is not positioned at a level horizontal with the eyes | Kanchanomai S et al,2011 | 1.640 | 1.130, 2.360 |
| Desks and chairs not matching height | Tian ZY et al,2019 | 1.951 | 1.009, 3.773 |
| Many social media | Ayhualem S et al,2021 | 2.546 | 1.360, 4.764 |
| Major | Lin YZ et al,2022 | 1.148 | 1.018, 1.296 |
| The years of smartphone use | Wah SW et al,2022 | 5.01 | 1.12, 22.38 |
| Top-level sport | Hodacová L et al,2022 | 21.47 | 1.84, 250.92 |
